# Anticancer Role of PPAR*γ* Agonists in Hematological Malignancies Found in the Vasculature, Marrow, and Eyes

**DOI:** 10.1155/2010/814609

**Published:** 2010-02-28

**Authors:** P. J. Simpson-Haidaris, S. J. Pollock, S. Ramon, N. Guo, C. F. Woeller, S. E. Feldon, R. P. Phipps

**Affiliations:** ^1^Department of Medicine/Hem-Onc Division, School of Medicine and Dentistry, University of Rochester, Rochester, NY 14642, USA; ^2^Department of Microbiology and Immunology, School of Medicine and Dentistry, University of Rochester, Rochester, NY 14642, USA; ^3^Department of Pathology and Laboratory Medicine, School of Medicine and Dentistry, University of Rochester, Rochester, NY 14642, USA; ^4^Department of Environmental Medicine, School of Medicine and Dentistry, University of Rochester, Rochester, NY 14642, USA; ^5^Department of Opthalmology, School of Medicine and Dentistry, University of Rochester, Rochester, NY 14642, USA; ^6^The Lung Biology and Disease Program, School of Medicine and Dentistry, University of Rochester, Rochester, NY 14642, USA

## Abstract

The use of targeted cancer therapies in combination with conventional chemotherapeutic agents and/or radiation treatment has increased overall survival of cancer patients. However, longer survival is accompanied by increased incidence of comorbidities due, in part, to drug side effects and toxicities. It is well accepted that inflammation and tumorigenesis are linked. Because peroxisome proliferator-activated receptor (PPAR)-*γ* agonists are potent mediators of anti-inflammatory responses, it was a logical extension to examine the role of PPAR*γ* agonists in the treatment and prevention of cancer. This paper has two objectives: first to highlight the potential uses for PPAR*γ* agonists in anticancer therapy with special emphasis on their role when used as adjuvant or combined therapy in the treatment of hematological malignancies found in the vasculature, marrow, and eyes, and second, to review the potential role PPAR*γ* and/or its ligands may have in modulating cancer-associated angiogenesis and tumor-stromal microenvironment crosstalk in bone marrow.

## 1. Introduction

Peroxisome proliferator activated receptors (PPARs) are a subfamily of the larger nuclear hormone receptor superfamily of transcription factors [1, 2]. Three distinct but closely related isoforms designated PPAR*α*, PPAR*β*/*δ*, and PPAR*γ* make up the family. PPAR*γ* functions are further delineated by two isoforms PPAR*γ*1 and PPAR*γ*2, which arise due to alternative promoter usage accompanied by alternative splicing and/or polyadenylation of the primary transcript (recently reviewed in [3]). PPARs are best known for their roles in lipid homeostasis and energy metabolism including cholesterol and triglyceride turnover [4], obesity [5], metabolic syndrome [6–9], and diabetes [5, 10, 11]; however, since their discovery, the PPARs and/or PPAR agonists have been implicated in a broader spectrum of biological processes playing protective and homeostatic roles such as promoting wound healing [12, 13] and, for the most part, countering the effects of aging [14], cardiovascular disease [15, 16], inflammation and immune responses [17–19], thrombosis and hemostasis [7, 8, 17–21], pathological angiogenesis [22–32], and cancer [24, 25, 31–41]. 

A number of naturally occurring ligands activate PPAR*γ* (Table 1), such as unsaturated fatty acids and eicosanoids [42], 15-deoxy-Δ-12-14-prostaglandin J_2_ (15d-PGJ_2_), and components of oxidized low density lipoproteins (LDLs) [43]. The affinity of PPAR*γ* for many of the endogenous ligands is low and, in some cases the physiological relevance of the ligand needs to be determined. However, it is well accepted that 15d-PGJ_2_ is the most potent endogenous ligand for PPAR*γ*. The thiazolidinediones (TZDs) are a class of synthetic ligands with high affinity for PPAR*γ* that are used for their antidiabetic effects to sensitize cells to insulin [44]. Nonsteroidal anti-inflammatory drugs such as ibuprofen and indomethacin are low affinity PPAR*γ* ligands [45]. Furthermore, the synthetic triterpenoid, 2-cyano-3,12-dioxooleana-1,9-dien-28-oic acid (CDDO), and derivatives are high affinity ligands for PPAR*γ* [46] (Table 1). 

Two overarching principles should be kept in mind when weighing the plethora of therapeutic benefits touted for PPAR*γ* agonists. First, PPAR*γ* agonists evoke both PPAR*γ*-dependent and PPAR*γ*-independent effects, thus therapeutic benefits ascribed to certain PPAR*γ* ligands do not necessarily require interaction with the PPAR*γ* ligand binding domain. Although PPAR*γ*-independent effects induced by 15d-PGJ_2_ and CDDO are due in part to the electrophilic nature of these ligands [47–50], PPAR*γ*-independent effects induced by TZDs are through a number of signaling pathways including inhibition of Bcl-2/Bcl-xL function, proteasomal degradation of cell cycle- and apoptosis-regulatory proteins, and transcriptional repression [51]. Second, PPAR*γ* agonists have been shown to have paradoxical physiological effects, likely due to tissue-specific and/or context-dependent regulatory signaling events.

Recently, we reviewed the role of PPAR*γ* and its ligands in the treatment of hematological malignancies, which is summarized in Tables 1 and 2 [3]. The purpose of this paper is twofold: first to highlight the potential uses for PPAR*γ* agonists in anticancer therapy with special emphasis on their role when used as adjuvant or combined therapy in the treatment of hematological malignancies, and second, to review the potential role PPAR*γ* and PPAR*γ* ligands may have in modulating cancer-associated angiogenesis and tumor-stromal microenvironment crosstalk in bone marrow—two pathophysiological events associated with most all types of cancer including hematological malignancies.

## 2. Tumor-Stromal Microenvironment Crosstalk and Tumor-Associated Angiogenesis

### 2.1. Cancer Stem Cell Theory and Tumor Dormancy

A key issue of debate in cancer biology is whether tumor growth is caused by a substantial proportion of the tumor cells or exclusively by an infrequent subpopulation of cells termed cancer stem cells (CSCs) [52]. Regardless of the cancer type, most patients who have experienced many years of disease-free survival after successful treatment of the primary tumor ultimately die from metastatic disease. Patients who relapse must harbor cancer cells for years or even decades until the cancer cells overcome the regulatory mechanisms that keep the tumor in check. Dormant cancer cells are defined by a prolonged absence of or a balance in either proliferation or apoptosis, resulting in essentially a perpetual state of quiescence that protects them from conventional cytotoxic drugs, which only target actively proliferating cells. It is unknown whether dormant cancer cells represent a specialized subpopulation of cells programmed to stay dormant, an unspecialized population of cells not able to grow in the new microenvironment, or a combination of both [53]. CSCs are usually slowly cycling cells and thus insensitive to cytotoxic drugs as well [54, 55]. Dormant cancer cells are inferred to be CSCs or tumor initiating cells, as some prefer to call them [56]. Nonetheless, the relative frequency of CSCs varies as a function of both the tumor type and the specific experimental system used [57]. To date, published data most strongly support the presence of CSCs in hematologic malignancies such as leukemia [58], and in three major solid tumor types, including aggressive brain, breast, and colon cancers [59, 60]. Moreover, the existence of treatment resistant tumor cells following disease relapse has bolstered the theory that CSCs exist [56]. Thus, new approaches to target CSCs are actively being sought. 

Although little evidence is available to suggest whether PPAR*γ* agonists could be used to specifically target CSCs while sparing normal hematopoietic stem cells, a few studies have been reported. Chearwae and Bright [61] demonstrated that PPAR*γ* agonists inhibit the proliferation of brain CSCs by inducing cell cycle arrest and apoptosis, which was associated with upregulated expression of PPAR*γ* and inhibition of signal transducer and activator of transcription (Stat)-3 signaling. Saiki and colleagues [62] showed that pioglitazone inhibits the growth of human leukemia cell lines and primary leukemia cells while sparing normal stem cells. Preclinical testing has identified additional cancer therapeutics that selectively target leukemic stem cells but not normal stem cells, including idarubicin with the proteasome inhibitor, parthenolide (known as feverfew), and TDZD-8 [63]. These agents target the NF-*κ*B pathway, a critical link in the well-established association between inflammation and carcinogenesis. In that PPAR*γ* agonists inhibit both NF-*κ*B- and Stat3-mediated transactivation of target genes and both of these transcription factors play a prominent role in cancer progression (see Section 2.8 and references therein), it is a likely extension to consider a role for PPAR*γ* agonists to target CSCs.

### 2.2. Tumor-Associated Angiogenesis

**Figure 1:**
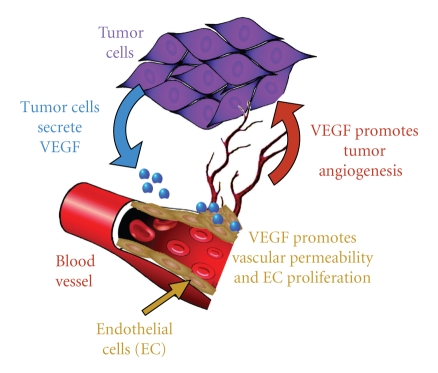
*Molecular mechanisms of tumor-associated angiogenesis.* Angiogenesis is essential for the persistence of solid tumor growth and, only recently, has it been appreciated that angiogenesis plays a role in progression of hematological malignancies as well. Cancer-associated angiogenesis in solid tumors begins once the tumor mass reaches a critical size such that the hypoxic environment inside the tumor leads to cancer cell-specific expression of proangiogenic factors including VEGF to shift the balance from endogenous antiangiogenic factors to tumor supplied proangiogenic factors—the angiogenic switch. Once proangiogenic factors overwhelm antiangiogenic factors, new blood vessels form in response to VEGF-induced endothelial permeability by EC sprouting, migration into the tumor mass, and proliferation from existing blood vessels—molecular mechanisms also induced by VEGF [64–67]. The tumor integrity of the vasculature is compromised in that it remains leaky with poor cell-to-cell adhesion, is abnormally branched and not well supported by pericytes (mural cells), the vascular smooth muscle cells that stabilize normal blood vessels [67, 68]. The chronic immaturity of tumor vessels has led Dvorak to characterize a tumor as a “wound that never heals” [69]. Notwithstanding, these features make tumor vessels viable targets for antitumor therapies. Benjamin et al. [70] demonstrated that removal of growth factors leads not only to the cessation of new vessel growth, but also to regression of the immature tumor vasculature [71].

Regardless of the type of cancer, once a primary tumor becomes established, it needs to develop its own blood supply for nutrient delivery and removal of toxic waste. The process of angiogenesis, that is the formation of new blood vessels from existing vasculature, involves complex interplay among cancer and stromal cell-secreted factors, extracellular matrix (ECM) constituents, and endothelial cells (ECs) (Figure 1). The adult vasculature is composed of quiescent ECs lining blood vessels and, with the exception of reproduction; the process of angiogenesis begins only in response to a broad array of tissue injury. 

Several isoforms of VEGF-A/165 are produced by alternative mRNA processing of the primary transcript, and these isoforms differ primarily in their ability to adhere to heparin or heparan sulfate proteoglycans (HSPGs) found both in the ECM or on the surface of stromal and tumor cells [72]. The VEGF gene family encodes VEGF isoforms A-F and placenta growth factor (PLGF) with at least three cognate receptors, VEGFR1/Flt-1, VEGFR2/Flk-1/KDR, VEGFR3/Flt-4 and two coreceptors, neuropilin (NRP) and HSPGs. VEGF-A/165 (hereafter designated VEGF) signaling through VEGFR2 is the major isoform responsible for pathological angiogenesis and induction of vascular permeability in tumors [73, 74], which leads to enhanced transendothelial migration of cancer cells during intravasation and extravasation [75]. VEGF-C and VEGF-D bind to VEGFR2 as well as VEGFR3 and are important for lymphangiogenesis and cancer metastasis to lymph nodes and spread through the lymphatic system [76–78]. 

VEGF production and intracrine signaling through VEGFR2 by ECs is essential for vascular homeostasis but is dispensable for angiogenesis as shown in EC-specific VEGF knockout mice [79]. Intracrine VEGF signaling requires expression of both VEGF and VEGFRs by the same cell and resistance to VEGF inhibitors that fail to penetrate the intracellular compartment. Hematopoietic stem cell survival also involves a VEGF-dependent internal autocrine loop mechanism [80]. Although it was originally thought that VEGFR expression was restricted to ECs, it is now apparent that other cell types express functional VEGFRs. Furthermore, VEGF is an autocrine growth factor for VEGFR-positive human tumors, including Kaposi sarcoma, melanoma, breast, ovarian, pancreatic, thyroid and prostate carcinomas, and leukemia [81–87]. Thus, in VEGFR-expressing tumors, VEGF inhibition may directly inhibit tumor cell growth as well as tumor-associated angiogenesis [83]. A host of proangiogenic factors play a role in pathological angiogenesis [64]; however, since most anticancer therapeutic strategies target the VEGF signaling pathway [64, 88], this paper focuses thereon.

### 2.3. Tumor-Stromal Microenvironment

**Figure 2:**
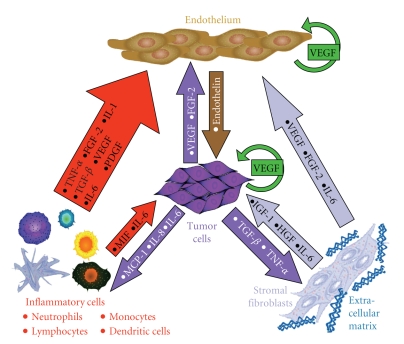
*Tumor-associated angiogenesis is sustained through stromal microenvironment crosstalk.* Most tumors are associated with the activation of tumor-promoting innate immune responses involving neutrophils, macrophages, and NK cells. Specific (adaptive) antitumor immune responses involving T- or B-lymphocytes are less efficient in suppressing tumor growth. Increased formation of blood and lymphatic vessels in bone marrow and lymph nodes provide oxygen and nutrients to malignant cells. Stromal cells, including ECs, inflammatory cells, and fibroblasts/myofibroblasts, produce cytokines and growth factors that act in a paracrine fashion to promote malignant cell proliferation or survival. In turn, malignant cells produce angiogenic factors and express their cognate receptors establishing functional autocrine loops to perpetuate their survival including signaling through the VEGF pathway [85–87, 107]. The secreted factors produced by and in response to those secreted by stromal and tumor cells include, but are not limited to VEGF, FGF-2, PDGF, IGF-1, HSF, TGF-*α*, TGF-*β*, TNF-*α*, IL-8, MCP-1/CCL2, MIF, IL-6, and IL-1 [95]. The potent vasoconstrictor peptide endothelin-1 has been implicated in the pathophysiology of atherosclerosis and its complications [108], as well as tumor angiogenesis and lymphangiogenesis [109, 110]. Proteases important for invasion thorough the basement membrane and remodeling of the ECM, such as plasminogen [96] and MMPs, including MMP-2 and MMP-9 [97], and their inhibitors, PAI-1/2 and TIMPs, respectively, are produced by stromal and tumor cells. Downregulation of endogenous inhibitors of angiogenesis such as thrombospondin (TSP)-1 occurs in the stromal compartment as well to favor angiogenesis, cancer cell growth, and metastasis [98]. In recent years, it has been recognized that a better understanding of the tumor-stromal microenvironment crosstalk may lead to elucidation of new therapeutic strategies for cancer therapy [99–102].

Paget's “seed and soil” hypothesis emphasizes the importance of the interaction between the tumor cell (“seed”) and its environment (“soil”) for metastasis to occur (reviewed in [89]). The stroma of the tumor microenvironment consists of several components including growth factors, chemokines, matrix glycoproteins and proteoglycans, proteases, and host cells that influence the behavior of cancer cells (reviewed in [90–102]). Host ECs, pericytes, macrophages, dendritic cells, lymphocytes, adipocytes, and fibroblasts/myofibroblasts present in the tumor microenvironment participate in the metastatic process (Figure 2). Initiation of new blood vessel formation requires activation of matrix metalloproteinases (MMPs) leading to degradation of the basement membrane, sprouting of ECs, and regulation of pericyte attachment for vessel stabilization. Activated fibroblasts, myofibroblasts, play an important role in synchronizing these events [94]. Furthermore, the topography of the ECM mediates vascular development and regulates the speed of cell migration during angiogenesis [103].

Chronic inflammation is associated with cancer initiation and progression [104–106]. Vascular ECs play a pivotal role in regulating leukocyte recruitment during inflammation [90]. Thus, in most cases, cancers exploit inflammation and recruited inflammatory cells for their own benefit [91]. Although activated inflammatory cells in the tumor microenvironment play important roles in cancer initiation, progression, angiogenesis, and metastasis [92], they are not the most numerous. Cancer-associated fibroblasts, which resemble myofibroblasts of healing wounds, are the most abundant cells of the tumor stroma [93], and contribute significantly to chronic inflammation, production of proangiogenic factors, and metastasis [94].

### 2.4. Inhibitors of Angiogenesis

As discussed above, angiogenesis is the hallmark pathology in tumor growth, progression, and metastasis. Inhibiting tumor angiogenesis adds to the arsenal of treatment options for a number of solid tumor types [111, 112], and recently has been proposed for hematological malignancies as well [107, 113–119]. Endogenous inhibitors of angiogenesis are critical for tight regulation of pathological angiogenesis; however, in response to malignant transformation the putative “angiogenic switch” bypasses this tight regulation to promote tumor progression [120]. Whereas radiation and chemotherapy target killing of the tumor cells, antiangiogenic therapy is primarily directed against tumor blood vessels. Endostatin [121, 122], angiostatin [122], and TSP-1 [123] are among a host of well-known endogenous inhibitors of angiogenesis [98, 124]. TSP-1 is a large molecular weight glycoprotein that inhibits the proliferation and migration of ECs by interacting with CD36 expressed on the cell surface; CD36 is a PPAR*γ* target gene. Small molecules based on a CD36-binding peptide sequence from TSP-1 are being tested for cancer treatment. One analog, ABT510, exhibits potent proapoptotic activity in vitro, while clinically it is very well tolerated with therapeutic benefits against several malignancies reported in phase II clinical trials [125–129].

Targeting VEGF-induced angiogenesis is in current use as monotherapy or combination therapy to treat a wide variety of cancers [130–132]. Bevacizumab (Avastin) and ranibizumab (Lucentis) are FDA-approved humanized monoclonal antibodies that recognize and block VEGF signaling in cancer and age-related macular degeneration (AMD) [130–134]. Additional, but not all-inclusive VEGF inhibitors (direct or indirect) are the RNA aptamer, pegaptanib; VEGF receptor decoy, VEGF-Trap (Aflibercept); small interfering RNA-based therapies, bevasiranib, and AGN211745; rapamycin, sirolimus; tyrosine kinase inhibitors including vatalanib, pazopanib, imatinib (Gleevec), TG100801, TG101095, AG013958, and AL39324; soluble VEGFRs; proteasome inhibitors, bortezomib (Velcade); thalidomide and derivatives. 

At present, established therapies have been very successful in reducing the vision loss associated with AMD [135, 136]; however, a number of reports on the clinical outcomes of antiangiogenic therapy with VEGF inhibitors have shown equivocal results [88, 137–141]. Unfortunately, no significant survival benefit has been demonstrated in anti-VEGF monotherapy trials. When anti-VEGF inhibitors are used in combination with standard chemotherapeutic approaches for solid tumors, such treatment does not prolong survival of cancer patients for more than a few months [137–141], except as shown in phase II and phase III clinical trials for metastatic colon cancer and metastatic breast cancer where median survival over chemotherapy alone was extended ~15–26 months (reviewed in [142]). Although different classes of VEGF-targeted therapies inhibit primary tumor growth, recent studies surprisingly report that treatment with VEGF inhibitors leads to more invasive and metastatic tumors [139, 143]. Most patients who initially respond to VEGF-targeted therapy will develop resistance, and the molecular and cellular mechanisms promoting resistance are poorly understood [137, 138]. Thus, resistance or refractoriness of tumor ECs to treatment with VEGF inhibitors limits the utility of long-term treatment [143]. These findings indicate that new studies and molecular approaches are needed to overcome the lack of sensitivity or resistance of tumor ECs to antiangiogenic therapies.

### 2.5. Targeting Transcription Factor Signaling Pathways Activated in Angiogenesis

Although VEGF is upregulated in response to many inducers activated in cancer, only two major transcription factors have been identified for its promoter, hypoxia inducible factor (HIF)-1 and Stat3 [144]. Both HIF-1 production and Stat3 activity are upregulated in many types of cancer. VEGF is strongly induced by the hypoxic tumor microenvironment before the tumor becomes vascularized, and thus, is important in hypoxic regulation of angiogenesis [145, 146]. HIF-1 is composed of the constitutively expressed HIF-1*β* subunit (aka the aryl hydrocarbon nuclear translocator/ARNT [146]) and an O_2_- and growth factor-regulated HIF-1*α* subunit. HIF-1*α* is also constitutively expressed but rapidly degraded under normoxia due to hydroxylation at two proline residues within the central degradation domain. Hydroxylation increases the affinity of HIF-1*α* for the tumor suppressor protein von Hippel-Lindau (pVHL) E3 ligase complex, which mediates ubiquitination and proteasomal degradation of HIF-1*α* thereby preventing formation of an active HIF-1 heterodimer [147]. Because the HIF hydroxylases have an absolute requirement for oxygen, hydroxylation is suppressed under hypoxic conditions allowing the HIF-1*α* subunit to accumulate, translocate to the nucleus, and heterodimerize with HIF-1*β* to activate transcription of target genes [148]. 

Activation of the Jak/Stat3 pathway by IL-6 through its high affinity receptor, IL-6R*α*, and its binding partner, gp130, is a well-known inflammatory response evoked by the acute phase response of innate immunity [149, 150]. Stat3 is a latent transcription factor whose maximal activation requires both tyrosine (Y-705) and serine (S-727) phosphorylation. Inhibition of Stat3 activation blocks HIF-1 and VEGF expression in vitro and inhibits tumor growth and angiogenesis in vivo [151]. Activation of Stat3 signaling by various mitogens is prevalent in different types of cancers. Furthermore, when Stat3 is inhibited, tumor cells will no longer express proangiogenic mediators in response to IL-6R signaling. Because Stat3 is constitutively active in many types of cancers, it is considered oncogenic [152, 153]. Therefore, Stat3 is an apt upstream target for inhibiting tumor VEGF expression and angiogenesis [151].

NF-*κ*B transcription factor links inflammation and tumorigenesis, and its activation allows both premalignant and malignant cells to escape apoptosis [154]. NF-*κ*B signaling occurs in essentially all aspects of cancer progression from uncontrolled growth, evasion of apoptosis, tumor cell invasion through stromal compartments and into the blood stream, and sustained angiogenesis [104, 154]. Constitutive NF-*κ*B activation is found in lymphoid and myeloid malignancies, including preneoplastic conditions, emphasizing its role in malignant transformation [155, 156]. More than 200 genes involved in cell survival, apoptosis, cell growth, immune responses and inflammation are transactivated by NF-*κ*B [157]. NF-*κ*B is sequestered in the cytoplasm by inhibitor proteins such as I*κ*B*α* [104, 154–156]. Upon activation, proteasomal degradation of I*κ*B*α* releases NF-*κ*B, which then translocates to the nucleus to bind to the *κ*B response element in promoter regions of target genes. Thus, small inhibitory molecules that target these various steps are continually being sought for cancer treatment. PPAR*γ* agonists have anti-inflammatory properties that are conferred, in part, through their ability to inactivate transcription factors that regulate inflammation including Stat3, NF-*κ*B, and AP-1 [158–160]. The potential for PPAR*γ* agonists as inhibitors of Stat3 and NF-*κ*B survival signaling in hematological malignancies is discussed in Section 2.8.

### 2.6. Angiogenesis and Targeted Antiangiogenic Therapy in Hematological Malignancies

Since hematological malignancies originate in bone marrow and lymphatic organs and do not form solid tumor masses, it was generally believed that angiogenesis would not be as critical for cancer progression as in solid tumors. In the recent years, however, the importance of angiogenesis and lymphangiogenesis in hematological malignancies has been recognized and discussed in detail in a number of excellent reviews and references therein [113, 114, 116–119]. Because PPAR*γ* agonists are being tested as inhibitors of angiogenesis, it is important to understand the role of angiogenesis and associated signal transduction pathways in the progression of hematological malignancies. Increased bone marrow microvessel density (MVD), an in vivo measure of tumor-associated angiogenesis, is found in hematological malignancies [161], confirming the importance of angiogenesis for malignant progression. 

In general, increased MVD correlates with increased disease burden and poor prognosis or treatment outcome [118]. A number of antiangiogenic agents have been used to treat hematological malignancies as discussed in the review articles cited above. For example, thalidomide, well known as a potent teratogen causing stunted limb growth, has gained favor as an inhibitor of angiogenesis in multiple myeloma (MM) [162–167]. Thalidomide and similar immunomodulatory drugs and proteasome inhibitors (e.g., bortezomib) exert their effects directly by induction of apoptosis of MM cells or indirectly by inhibiting production of cytokines and proangiogenic factors, including VEGF, by bone marrow stromal cells (BMSCs) [162, 168]. The angiogenic activity of MM ECs correlates with downregulated expression of the endogenous antiangiogenic factor, endostatin [169]. Increased MVD in bone marrow correlates with shorter overall disease-free survival in AML, and elevated VEGF mediates both autocrine and paracrine signaling in support of leukemia cell survival and induction of angiogenesis [86, 87, 113, 161]. 

Angiogenesis in chronic lymphocytic leukemia (CLL) occurs in both marrow and lymph nodes [170]. Increased vascularity leads to elevated production of hematopoietic growth factors by new vessel ECs, which stimulates expression of VEGF and VEGFRs by CLL cells for autocrine signaling to promote survival [113, 170]. Elevated levels of VEGF are found in the serum of patients with chronic myeloid leukemia (CML), which correlates with worse survival [171]. Non-Hodgkin lymphoma (NHL) cells secrete VEGF and express VEGFRs, which also contribute to autocrine and paracrine signaling [172]. A phase II clinical trial of bevacizumab (Avastin) therapy in patients with relapsed, aggressive NHL showed a median increase in disease-free survival by 5.2 months [115], suggesting that anti-VEGF therapy is a limited but viable target for treatment. Antiangiogenic therapy would likely be more efficacious if combined with active chemotherapy regimens [115, 173]. Increased MVD in lymph nodes and elevated VEGF are statistically correlated with a greater tumor burden in Hodgkin lymphoma in newly diagnosed patients [174, 175]. Survival after treatment of diffuse large-B-cell lymphoma is adversely affected in patients whose tumor stroma show elevated MVD, indicating that differences in the tumor microenvironment play a critical role in treatment outcomes [176]. However, the role of angiogenesis varies in lymphoma subtypes due to heterogeneity in expression of proangiogenic factors [113, 177]. 

In addition to agents targeting VEGF-VEGFR signaling directly, a number of agents have been developed to target the tumor microenvironment (reviewed in [99–102]), including ECM modulators, tyrosine kinase inhibitors, and immunomodulators, many of which indirectly target cancer angiogenesis. Nonetheless, autocrine VEGF signaling to promote malignant cell survival appears to be a common theme in hematological malignancies [85–87, 107, 113, 170, 172, 178], suggesting that anti-VEGF/VEGFR targeted therapy would promote direct killing of tumor cells, as well as inhibit angiogenesis associated with several types of hematological malignancies. It should be noted that antiangiogenic therapy in combination with conventional therapy for metastatic colon cancer and metastatic breast cancer significantly increased survival [142]; these cancers represent two of the three solid tumors (the third being brain cancer) for which published data most strongly support the presence of CSCs [59, 60]. In that CSCs have been documented in hematologic malignancies such as leukemia [58], it is interesting to speculate that patients with hematological malignancies other than leukemias may benefit from adding antiangiogenic therapy to standard treatments if CSCs could be identified in the malignant population of cells.

### 2.7. Effects of PPAR*γ* and PPAR*γ* Ligands on EC Functions and Angiogenesis

The endothelium releases a balance of bioactive factors that regulate vasoconstriction and relaxation to facilitate vascular homeostasis [179]. During homeostasis, the endothelium also inhibits platelet and leukocyte adhesion to the vascular surface and maintains the balance between prothrombotic and profibrinolytic activities. Several common conditions with a predisposition to atherosclerosis, including hypercholesterolemia, hypertension, diabetes, and stroke, are associated with endothelial dysfunction, leading to a proinflammatory and prothrombotic endothelium [180]. For more than a decade investigators have studied the effects of PPAR*γ* ligands on EC functions with a particular interest in determining whether they could be used to inhibit cancer cell growth (reviewed in [25, 31, 181, 182]) and cancer-associated angiogenesis (reviewed in [23, 25, 31, 181–184]). The functions that PPAR*γ* ligands target during angiogenesis include induction of apoptosis, inhibition of EC proliferation, downregulation of proangiogenic factors, and as inhibitors of the inflammatory events that trigger and perpetuate pathological angiogenesis (Table 3). In addition to targeting tumor angiogenesis, PPAR*γ* ligands have direct effects on cancer cells due to their ability to promote apoptosis, inhibit cell proliferation or induce differentiation [3, 71, 185–188]. However, to date, disappointing results have been obtained in phase II clinical trials using the PPAR*γ* ligand troglitazone to inhibit progression of treatment-refractory metastatic breast cancer [189], chemotherapy-resistant metastatic colorectal cancer [190], and prostate cancer [191]. In recent years, the focus has shifted from treating the tumor to targeting the signaling pathways that drive aberrant cell proliferation and survival and tumor-associated angiogenesis. Such targets have the potential for greater specificity together with reduced systemic toxicity [104].

### 2.8. Therapeutic Potential of PPAR*γ* and PPAR*γ* Ligands to Target Angiogenic Signaling Pathways in Treatment of Hematological Malignancies

It has been suggested that PPAR*γ* functions as a tumor suppressor gene [204]; therefore, it is important to understand the complexity of signal transduction pathways and molecular players affected by PPAR*γ* that promote tumor growth, cancer-associated angiogenesis, and metastasis. MM, a progressive hematological malignancy of plasma cells, remains largely incurable with survival averaging 3–5 years despite conventional and high-dose therapies; therefore, novel treatment approaches are desperately needed. MM is characterized by excessive numbers of abnormal plasma cells in the bone marrow and overproduction of intact monoclonal immunoglobulin (IgG, IgA, IgD, or IgE) or Bence Jones protein (free monoclonal *κ* and *λ* light chains). Common clinical manifestations of MM are hypercalcemia, anemia, renal damage, increased susceptibility to bacterial or viral infection, and impaired production of normal immunoglobulins (http://www.themmrf.org/living-with-multiple-myeloma/newly-diagnosed-patients/what-is-multiple-myeloma/definition.html). Lytic lesions are often found in the bone including the pelvis, spine, ribs, and skull. Furthermore, neovascularization in bone marrow parallels disease progression of MM [205]. 

Our laboratory has shown that normal and malignant B cells, including MM, express PPAR*γ* [206–210], and that certain PPAR*γ* ligands can induce apoptosis in MM cells [207, 208]. Because PPAR*γ* ligands also have PPAR*γ*-independent effects, we examined the functional consequences of PPAR*γ* overexpression in human MM [207]. PPAR*γ* overexpression in myeloma cells decreased cell proliferation, induced spontaneous apoptosis even in the absence of exogenous ligand, and enhanced their sensitivity to PPAR*γ* ligand-induced apoptosis. Apoptosis was associated with the downregulation of anti-apoptotic proteins XIAP and Mcl-1 as well as induction of caspase-3 activity [207]. IL-6 mediates growth and survival of human myeloma cells through the MEK/MAPK and Jak/Stat signaling pathways, and IL-6 confers protection against dexamethasone-induced apoptosis via activation of the protein tyrosine phosphatase, SHP2 [211]. Binding of MM cells to BMSCs triggers expression of adhesive molecules and secretion of IL-6, promoting MM cell growth, survival, drug resistance, and migration. Furthermore, PPAR*γ* overexpression-induced cell death of myeloma cells is not abrogated by coculture with BMSCs [207]. Overexpression of PPAR*γ* in myeloma cells and BMSCs inhibited both basal and myeloma cell adhesion-induced IL-6 production by BMSCs. These results indicate that PPAR*γ* negatively controls MM growth and viability, in part, through inhibition of IL-6 production by BMSCs [207]. Wang et al. [211] showed that myeloma cells express PPAR*γ* and that the PPAR*γ* agonists, 15d-PGJ_2_ and troglitazone, abolish IL-6-inducible myeloma cell proliferation and promote apoptosis in a PPAR*γ*-dependent manner. These PPAR*γ* agonists also reduced cell-cell adhesion between BMSCs and MM cells and overcame resistance to dexamethasone-mediated apoptosis in the MM.1R cell line through a PPAR*γ*-dependent mechanism [212]. Taken together, the results of these studies demonstrate that PPAR*γ* agonists can be used to inhibit IL-6-dependent crosstalk between myeloma cells and BMSCs [207, 211, 212], validating novel therapeutic strategies that target the tumor-stromal microenvironment. 

Dankbar and colleagues [205] demonstrated that biologically active VEGF is expressed and secreted by myeloma cell lines and plasma cells isolated from the marrow of patients with MM. However, the myeloma cells did not express or only weakly expressed VEGFR1 and VEGFR2, indicating that autocrine VEGF signaling in MM is unlikely. In contrast, they demonstrated that BMSCs abundantly express VEGFR2 and that such expression could be stimulated in response to IL-6. In addition, exposure of BMSCs and microvascular ECs to VEGF induced a time- and dose-dependent increase in IL-6 secretion. They showed that IL-6-stimulated VEGF expression in and secretion from myeloma cell lines and in plasma cells purified from the marrow of patients with MM as well. Thus, this study confirms that paracrine interactions between myeloma and marrow stromal cells triggered by VEGF and IL-6 represent feasible signal transduction pathways to target for treatment of MM [205].

**Figure 3:**
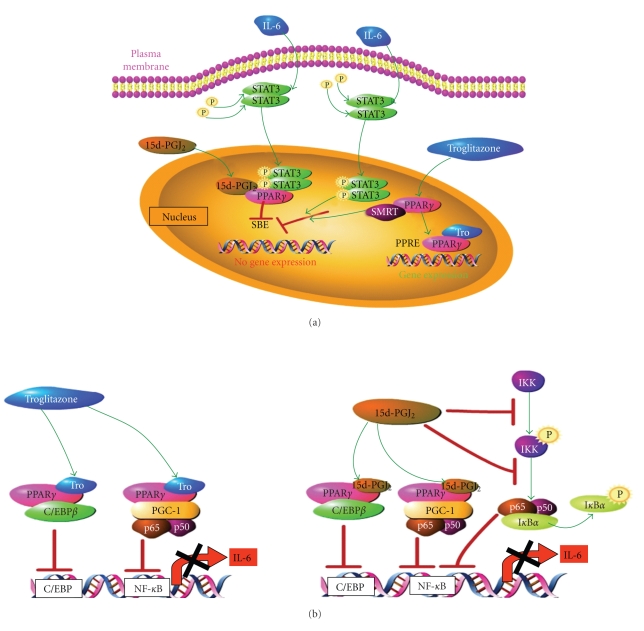
*PPAR*γ* agonists inhibit Stat3-mediated IL-6 gene expression in myeloma cells.* Inactivation of IL-6-activated Stat3 by PPAR*γ* agonists occurs in a PPAR*γ*-dependent manner; however, the molecular mechanisms by which two distinct PPAR*γ* agonists (15d-PGJ_2_ and troglitazone) suppress IL-6-activated Stat3 in MM cells differ as shown in (a) [211]. Direct complex formation between phosphorylated Stat3 and PPAR*γ* activated by 15d-PGJ_2_ prevents Stat3 binding to its cognate response element (SBE) on the promoters of target genes ((a), left). This mode of transcriptional inactivation does not require binding of the activated PPAR*γ* transcription factor to DNA in the promoter region and, thus, can occur in the absence of a PPRE. However troglitazone activated PPAR*γ* promotes redistribution of the corepressor SMRT from PPAR*γ* to phosphorylated Stat3 so that Stat3 can no longer recruit the transcriptional machinery necessary for gene expression ((a), right) [211]. High levels of IL-6 are found in MM and promote myeloma cell proliferation and survival and indirectly promote tumor-associated angiogenesis. The PPAR*γ* agonists troglitazone and 15d-PGJ_2_ have been shown to inhibit transcription of the IL-6 promoter driven by C/EBP*β* and NF-*κ*B [212]. Troglitazone-activated PPAR*γ* binds to C/EBP*β* preventing binding to its cognate response element on the IL-6 promoter, which is the major mechanistic pathway of troglitazone-mediated downregulation of IL-6 expression. In addition activated PPAR*γ* competes with NF-*κ*B for the PGC-1 coactivator, which leads to decreased NF-*κ*B binding to the *κ*B response element on the IL-6 promoter contributing to inhibition of IL-6 gene expression, albeit to a lesser extent than inhibition of C/EBP*β* ((b), left). A slightly different mechanistic emphasis on PPAR*γ*-mediated inhibition of IL-6 gene expression occurs in response to 15d-PGJ_*2*_. Although 15d-PGJ_2_-activated PPAR*γ* inhibits C/EBP*β*-mediated transactivation of the IL-6 promoter similarly to troglitazone-activated PPAR*γ*, the predominant mode of inhibition is through 15d-PGJ_2_-activated PPAR*γ* using the coactivator PGC-1 as a bridging protein to interact with NF-*κ*B to prevent transactivation of the IL-6 promoter. Furthermore, 15d-PGJ_2_ inactivates NF-*κ*B by inhibiting phosphorylation of IKK and I*κ*B independently of PPAR*γ* activation ((b), right). The schematics in this figure were adapted from [211, 212].

PPAR*γ* ligands are known to inhibit or repress the activity of a number of transcription factors important in innate immunity, inflammation and cancer, including Stat3 and NF-*κ*B [158, 159]; therefore, targeted inhibition of Stat3 and NF-*κ*B activity with PPAR*γ* agonists is a relevant avenue of investigation for new cancer therapeutics [213]. Wang and colleagues [211] showed that 15d-PGJ_2_ and troglitazone significantly inhibited Stat3 binding to its cognate response element and inhibited Stat3 binding to the promoters of c-MYC and MCL-1 thereby preventing transactivation of these Stat3 target genes. Whereas 15d-PGJ_2_ promotes direct binding of PPAR*γ* to Stat3 forming a complex such that Stat3 is no longer capable of binding to the type II IL-6 response element on promoters of Stat3 target genes, troglitazone induces the redistribution of the corepressor NCoR/SMRT from PPAR*γ* to Stat3, which leads to repression of Stat3 transactivation of target genes [211] (Figure 3(a)). In contrast, 15d-PGJ_2_ and troglitazone did not affect the expression of IL-6R or activation by phosphorylation of the downstream signaling molecules Jak/Stat3, MAPK, and PI3K/Akt in myeloma cells [211]. 

PPAR*γ* and its ligands effectively blocked IL-6 transcription and secretion from BMSCs that is induced in response to myeloma cell adhesion [212]. Such inhibition occurs through competition between PPAR*γ* and NF-*κ*B for the coactivator PGC-1, which is recruited from p65/p50 complexes by ligand-activated PPAR*γ* (Figure 3(b)). Direct complex formation between PPAR*γ* and C/EBP*β* also prevents transactivation of the IL-6 promoter. The natural PPAR*γ* ligand, 15d-PGJ_2_, has a PPAR*γ*-independent effect on NF-*κ*B by decreasing phosphorylation of IKK and I*κ*B to prevent activation of NF-*κ*B [212]. Prolonged treatment with the PPAR*γ* ligand CDDO-Me inactivates Erk signaling in AML cells effectively inhibiting cell growth [214]. In vitro studies show that CDDO-Me inactivates Stat3, Src, and Akt; reduces expression of the c-MYC gene; promotes accumulation of cells in the G2-M phase of the cell cycle; and, abrogates invasive growth and induction of apoptosis of mammary cells [215]. Furthermore, mammary cell growth and lung metastases were completely eliminated in mice treated with CDDO-Me starting one day after tumor implantation; tumor growth was significantly inhibited when started after 5 days. Thus, CDDO-Me may have therapeutic potential for hematological malignancies and solid tumors through inactivation of Stat3 [215].

Bortezomib (Velcade, formerly PS-341) is a proteasome inhibitor that is used for antiangiogenic therapy in various cancers including MM [216]. Bortezomib targets myeloma cells and also inhibits the binding of myeloma cells to BMSCs. Furthermore, intravenous bortezomib, with or without dexamethasone, is well tolerated and effective in treating patients with relapsed or refractory MM [216]. Because bone marrow angiogenesis plays an important role in the pathogenesis and progression of MM and bortezomib inhibits angiogenesis, Roccaro and colleagues [217] tested the effects of bortezomib on the angiogenic phenotype of MM patient-derived ECs (MMECs). At clinically relevant concentrations, bortezomib inhibited the proliferation of MMECs and human umbilical vein endothelial cells (HUVECs) in a dose-dependent and time-dependent manner. Bortezomib also inhibited angiogenesis as measured by capillary tube formation on Matrigel in vitro and in the chick embryo chorioallantoic membrane assay in vivo [217]. Furthermore, binding of drug sensitive MM cells (MM.1S) to MMECs triggered their proliferation, which was prevented by bortezomib. Bortezomib also triggered a dose-dependent inhibition of VEGF and IL-6 production by and secretion from MMECs and abrogated IL-6 triggered signaling cascades via caspase-dependent downregulation of gp130 in MM [218]; gp130 is the signaling component of the high affinity IL-6R complex that activates Stat3. These data provide mechanistic insight on the antiangiogenic effects of bortezomib on MMECs in the bone marrow microenvironment [217] and support the concept that adding antiangiogenic agents as adjuvant or combination therapy with standard therapy would be more efficacious in treating patients with relapsed or refractory MM [219], and perhaps other hematological malignancies as well.

 Although inhibiting IL-6 signaling through its high affinity receptor promotes apoptosis of MM cells when cocultured with BMSCs, some myeloma cells survive suggesting that the marrow microenvironment stimulates IL-6-independent pathways that exert a prosurvival effect [220]. BMSCs stimulate MAPK signaling in myeloma cells through IL-6R-independent mechanisms thereby circumventing the need for Stat3-mediated signaling in response to IL-6 for myeloma cell survival. Chatterjee et al. [220] went on to show that disruption of both the IL-6R/Stat3 and MAPK signaling pathways led to significantly more apoptosis of MM cell lines and primary MM cells even in the presence of BMSCs than singly inhibiting each signaling pathway. These results suggest that combined targeting of different and independently activated pathways is required to efficiently induce apoptosis of MM cells in the marrow microenvironment [220].

It should be kept in mind that anti-VEGF/VEGFR-targeted therapy could occur through a number of mechanistic pathways, such as direct inhibition of VEGF-induced angiogenesis or indirectly through mechanisms that inhibit expression of additional proangiogenic factors, promote apoptosis, or induce tumor dormancy [88, 221]. Rather than target the VEGF-signaling pathway, it may be possible to alter the phenotype of the angiogenic endothelium. The angiogenic EC phenotype is characterized by marked downregulation of CD36/fatty acid translocase (FAT) [222]. CD36 is a glycoprotein associated with normal and pathologic processes including scavenger receptor functions, lipid metabolism and fatty acid transport, cell adhesion, angiogenesis, modulation of inflammation, activation of TGF-*β*, atherosclerosis, diabetes, and cardiomyopathy [223]. PPAR*α* regulates expression of CD36 in mouse liver and PPAR*γ* regulates its expression in mouse adipose tissues [224, 225]. Furthermore, statins and PPAR*γ* ligands together have an additive effect on upregulation of CD36 production by potentiating the transcription of the CD36 gene in monocytes [226]. CD36 is the cellular receptor for TSP-1 on microvascular endothelium and is necessary for its antiangiogenic, proapoptotic activity, making CD36 an attractive target for development of therapeutic agents [227]. 

Vascular endothelium expression of CD36 is sporadic however, with lower levels of expression in larger vessels [196, 228]. As discussed in Section 2.4, loss of endogenous inhibitors of angiogenesis in favor of proangiogenic factors produced by tumors leads to tumor-associated angiogenesis. A small peptide (ABT510) derived from TSP-1 type 1 repeats binds to CD36 and blocks tumorigenesis by reversing the “angiogenic switch” [229]. Huang et al. [196] demonstrated that 15d-PGJ_2_, troglitazone, and rosiglitazone potentiate the antitumor activity of AB510 in a CD36-dependent manner. Furthermore, these ligands upregulated EC expression of PPAR*γ* and CD36 [43, 196], which likely leads to the synergistic inhibition of tumor-associated angiogenesis and induction of EC apoptosis in vivo [196]. Importantly, lower doses of PPAR*γ* agonists could be used in combination with AB510 to significantly reduce tumor-associated angiogenesis and promote EC apoptosis. This study provides compelling evidence that PPAR*γ* ligands could be useful as adjuvant or combination therapy in treatment of tumor angiogenesis.

Another important molecular mechanism to target for intervention of cancer progression in hematological malignancies is regulation of stromal matrix remodeling by proteases [193, 230]. PAI-1 production by ECs inhibits plasmin-mediated proteolytic degradation of the ECM. PPAR*γ* ligands upregulate expression and release of PAI-1 from ECs [193], which would inhibit degradation of tumor-associated fibrin leading to EC migration, proliferation, and angiogenesis [231]. PPAR*γ* ligands inhibit the adhesion of the myeloid leukemia HL-60 and K562 cells to the ECM as well as their invasion through Matrigel [230]. In addition, 15d-PGJ_2_ and troglitazone in both the HL-60 and K562 cell lines significantly inhibited MMP-9 and MMP-2 expression and proteolytic activities. The results of this study suggest that PPAR*γ* ligands may inhibit leukemic cell adhesion to and invasion through the ECM as well as regulate angiogenesis by inhibiting matrix remodeling that favors cancer cell invasion and EC migration [230].

### 2.9. MicroRNAs and PPAR*γ* Agonists in Hematological Malignancies

MicroRNAs (miRNAs) are short noncoding RNAs that function as negative regulators of the stability and/or translation of specific target mRNAs [232–234]. Typically, miRNAs target a cluster of genes instead of one specific gene, and a single miRNA can have more than 100 targets [233, 235]. Regulation of gene expression by miRNAs is increasingly being accepted as a pivotal point in cell function, either in normal development or disease states (recently reviewed in [234, 236–238]). Mature miRNAs derive from primary miRNA transcripts that are either transcribed from their own promoter regions [239] or processed introns spliced from pre-mRNAs [240]. Primary miRNAs are first processed in the nucleus by the RNase III endonuclease, Drosha, to form pre-miRNAs [241]. Pre-miRNAs are exported out of the nuclear compartment into the cytoplasm by exportin-5 [242]. Once in the cytoplasm, the pre-miRNA is further processed by another RNase III endonuclease, Dicer [243]. Finally, the mature miRNA is loaded onto the Argonaute (Ago) protein and incorporated into the ribonucleoprotein complex, RISC (RNA induced silencing complex) [244], which directs the miRNA to its target mRNA. Mature miRNAs primarily bind to transcripts through imperfect Watson-Crick base pairing to conserved miRNA binding sites in the 3′ untranslated region (UTR) of target mRNAs [234, 245]. The ability of miRNAs to regulate the expression of numerous genes at once often leads to pleiotropic effects and can modulate multiple cellular pathways. 

There is growing evidence that dysfunctional expression of miRNAs is a common feature of malignancy in general and hematological malignancy in particular [233, 246]. Aberrant miRNAs have been documented in almost all hematological malignancies [247]. For example, Calin and colleagues [248] first implicated miRNAs in hematological malignancies when they demonstrated that miR-15 and miR-16 are frequently deleted or downregulated in CLL associated with deletions on chromosome 13q14. Deletion or downregulated expression of miR-15a and miR-16 on chromosome 13 is also found in MM cells [249]; deletion of chromosome 13 predicts significantly reduced survival in patients with MM [250]. In 2005, another group reported that the polycistronic precursor transcript of the miR-17~92 cluster, which encodes seven different miRNAs, is overexpressed in human B cell lymphomas and acts as an oncogene [251]. The miR-17~92 cluster is amplified and/or overexpressed in other hematological malignancies including AML [252, 253] and MM [246], as well as cancers of epithelial origin such as lung [254], thyroid [255], and hepatocellular [256] carcinomas. Overexpression of miR-21 occurs in MM [246, 257] and other cancers including glioblastoma [258] and breast cancer [259]. Thus, there is enormous hope that miRNA research will provide breakthroughs in the understanding of cancer pathogenesis and in the development of new prognostic markers [260]. 

Kuehbacher et al. [261] recently reviewed miRNAs that possess proangiogenic or antiangiogenic function. The miR-17~92 cluster, let-7f, and miR-27b posses proangiogenic functions, in part, by inhibiting expression of TSP-1 and CTGF. A role for miR-221 and miR-222 in blocking angiogenesis is suggested by their ability to inhibit EC migration, proliferation, and angiogenesis in vitro. In addition, miR-21 is implicated in the invasive and metastatic properties of colon and breast cancer cell lines by targeting multiple tumor suppressor genes, such as PTEN, TPM1, and MASPIN [259, 262, 263]. Moreover, miR-21 overexpression, which occurs in MM as discussed below, is associated with advanced clinical disease, lymph node metastasis and poor prognosis for overall survival in breast cancer [264]. The Sessa group demonstrated that a functional miRNA biogenesis pathway is required for angiogenesis [265, 266]. Inactivation of Dicer, the miRNA processing enzyme, impairs angiogenesis induced by multiple stimuli such as VEGF, and during tumorigenesis and wound healing [266]. VEGF also induces the expression of several proangiogenic miRNAs including the miR-17~92 cluster [266]. Furthermore, miR-130a functions in angiogenesis by inhibiting expression of two antiangiogenic homeobox transcription factors, HOXA5 and GAX [267]. 

Although the mechanisms regulating expression of miRNAs are only beginning to be understood [234, 236–238, 268, 269], key regulators of the biosynthetic pathway are often abnormally expressed in hematological malignancies [270]. Recently, Löffler and colleagues [257] demonstrated that survival of IL-6-dependent MM cells involves Stat3-mediated induction of miR-21. Two bona fide IL-6 type II-response elements for Stat3 binding are located upstream of the miR-21 genes of various vertebrate species [257]. Stat3 regulates transactivation of several anti-apoptotic genes such as survivin, Bcl-2, and Mcl-1. Löffler et al. [257] suggest that Stat3 induction of miR-21 represents a “slow-acting yet long-lasting” survival stimulus to complement the immediate induction of anti-apoptotic proteins. The cancers in which miR-21 is overexpressed contain constitutively activated Stat3 for survival or growth [257]. These results suggest that miR-21 is important for the oncogenic potential of Stat3 in the pathogenesis of MM and other malignancies. IL-6-mediated activation of Stat3 is also important for transformation of nonmalignant breast epithelial cells to self-renewing mammospheres that contain CSCs [271]. Inflammation in cancer leads to elevated IL-6 production by two mechanisms: Src-mediated activation of NF-*κ*B leading to transactivation of the IL-6 gene, and rapid degradation of let-7 miRNA, which is a direct inhibitor of IL-6 expression [271]. Let-7 is downregulated in some cancers including Burkitt lymphoma [272] thereby leading to elevated IL-6 production, likely due to activation of the oncogenic NF-*κ*B-IL-6-Stat3 inflammatory pathway. In that the PPAR*γ* agonist CDDO-Me inactivates Src and Stat3 in cancer cells [215], further investigation of the efficacy of various PPAR*γ* ligands as anticancer agents is certainly warranted. 

Recently, Roccaro and colleagues [249] identified a multiple myeloma-specific miRNA signature characterized by downexpression of miR-15a and miR-16 and overexpression of miR-222, miR-221, miR-382, miR-181a, and miR-181b in bone marrow-derived CD138+ MM cells. Both miR-15a and miR-16 regulate proliferation and growth of plasma cells by inhibiting Akt and MAPK cell survival signaling pathways. However, both miR-15a and miR-16 are deleted on chromosome 13 associated with MM [249] thereby preventing normal repression of cell proliferation during cancer progression. Pichiorri et al. [246] also identified an miRNA signature associated with MM pathogenesis. Overexpression of miR-21, the miR-106b~25 cluster, and miR-181a and miR-181b was found in MM and monoclonal gammopathy of undetermined significance (MGUS) samples. On the other hand, selective upregulation of miR-32 and the miR-17~92 cluster was identified only in MM cells. Expression of suppressor of cytokine signaling (SOCS)-1, involved in negative feedback regulation of Jak/Stat signaling, is downregulated by miR-19a and miR-19b thereby leading to sustained IL-6-mediated MM cell proliferation [246]. Furthermore, miR-19a, miR-19b, miR-181a, and miR-181b antagonists suppress human MM tumor cell growth in nude mice, suggesting that miRNAs that modulate the expression of proteins critical to myeloma pathogenesis, including the IL-6-regulated Stat3 pathway, are potential targets for development of new therapeutic strategies for treatment [246]. 

**Figure 4:**
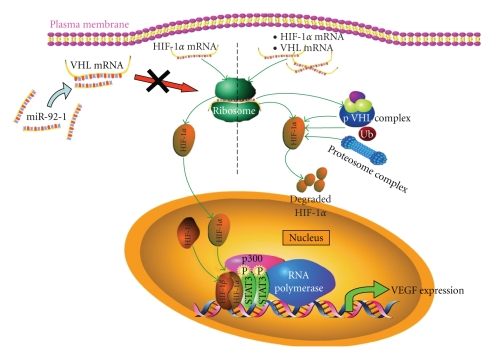
*Autocrine production of VEGF in CLL B cells is regulated by miRNA-92-1 inhibition of pVHL production.* Expression of high levels of VEGF by tumor cells is critical to promote and sustain the angiogenesis needed for cancer progression. Under normal oxygen tension, the HIF-1*α* subunit of the transcription factor, HIF-1, is constitutively produced and rapidly degraded by pVHL-induced proteasomal degradation, which prevents transcription of the VEGF gene. In solid tumors, HIF-1-induced VEGF expression occurs when tumor growth exceeds the dimensions where existing blood vessels can feed the tumor and carry away waste products. The resulting hypoxia leads to stabilization of HIF-1*α* and activation of the HIF-1 heterodimer resulting in high VEGF production by tumor cells. Although solid tumors do not develop in hematological malignancies, angiogenesis is an important process of disease progression. CLL B cells constitutively express high levels of VEGF and VEGFRs leading to autocrine signaling and increased resistance to apoptosis. Recently, Ghosh et al. [273] discovered that HIF-1 is stabilized in CLL B cells due to low levels of pVHL as a result of miR-92-1 overexpression and subsequent repression of translation of the VHL transcript. Therefore, HIF-1 accumulates and translocates to the nucleus where it forms an active complex with the transcriptional coactivator p300 and phosphorylated Stat3 and, together with the basal transcription machinery, transactivates the VEGF promoter. PPAR*γ* agonists could potentially inhibit overexpression of VEGF by inhibiting Stat3 signaling in CLL B cells. The schematic in this figure was adapted from [273].

The Stat3-regulated gene, HIF-1*α* *,* is constitutively expressed under normoxia in CLL B cells, most likely as a result of low production of pVHL, which is responsible for HIF-1*α* degradation. Ghosh and colleagues [273] demonstrated that overexpression of miR-92 in CLL B cells targets the VHL transcript to repress its translation (Figure 4). Furthermore, stabilized HIF-1*α* forms an active complex with the transcriptional coactivator p300 and activated Stat3 on the VEGF promoter, which likely explains the anomalous autocrine VEGF secretion from CLL B cells [273]. In that PPAR*γ* agonists inhibit the IL-6-regulated Stat3 signaling cascade, a role for PPAR*γ* agonists in regulating expression of miRNAs critical to the pathogenesis of hematological malignancies may be an important avenue of future scientific investigations. 

Recently, miRNAs have emerged as epigenetic regulators of metabolism and energy homeostasis [274]. It is clear that there is an obesity epidemic in the United States [275]. Increased body weight is associated with increased mortality for most all types of cancers including hematological malignancies [276]. Additional studies have confirmed that obesity puts patients at a moderate increased risk of developing MM [276–279], and that this risk may be higher in women than men [279]. An important link between obesity and MM is elevated expression of IL-6 in adipose tissue [280] and bone marrow [207], which also leads to elevations in circulating IL-6. Lin et al. [274] demonstrated that the miR-27 gene family is downregulated during adipogenic differentiation. Furthermore, overexpression of miR-27 specifically inhibits adipocyte formation and expression of PPAR*γ* and C/EBP*α*, the two master transcriptional regulators of adipogenesis. Although PPAR*γ* and C/EBP*α* mRNA and protein levels were markedly reduced by miR-27a or miR-27b, it was not a direct miRNA effect [274]. Expression of miR-27 is increased in fat tissue of obese mice and is regulated by hypoxia, an important extracellular stress associated with both obesity and cancer. During adipogenesis the expression of miR-27b, an important regulator of angiogenesis, is downregulated in human adipogenic stem cells, and PPAR*γ* mRNA expression increases concomitantly with decreasing miR-27b expression [281]. Both miR-27a and miR-27b directly bind RXR*α* mRNA and regulate RXR*α* translation in rat hepatic stellate cells [282]. It is well known that RXR*α* heterodimerizes with PPAR*γ* to activate numerous genes required for adipogenesis and energy metabolism. These data suggest that miR-27 represents a new class of adipogenic inhibitors and their downregulation may play a role in the pathological development of obesity [274]. Furthermore, in that PPAR*γ* is a master regulator of adipogenesis and target of insulin sensitizing drugs, it is reasonable to consider that the beneficial effects of PPAR*γ* agonists in cancer treatment may be exerted through regulation of energy homeostasis, at least in part, by modulation of miRNA expression. Indeed, the anticarcinogenic activity of one of the triterpenoids is due to repression of oncogenic miR-27a [283]. 

All known forms of the human PPAR*γ* mRNA contain numerous miRNA binding sites in the 3′UTR as predicted through different bioinformatic algorithm databases (TargetScan [284], miRanda [285], PicTar [286]). The miRNA binding sites for miR-27a/b, miR-130a/b, miR-301, miR-34a/b in the PPAR*γ* 3′UTR are conserved in human, chimp, mouse, and rat. Notably, two conserved binding sites for miRNAs in the PPAR*γ*  3′UTR are for miR-27b and miR-130a that have angiogenic or proliferative functions. It would be interesting to determine whether these miRNAs suppress PPAR*γ* function during angiogenesis and/or tumor growth. This could lead to identification of novel targets that may induce PPAR*γ* expression leading to the anticancer functions of cell differentiation and loss of proliferation. However, a role for PPAR*γ* agonists in miRNA-based therapeutic strategies to treat cancer awaits further clarification by new research endeavors.

## 3. Anticancer Role of PPAR*γ* Agonists as Adjuvant or Combination Therapy in Hematological Malignancies of the Eyes

### 3.1. Ocular Hematological Malignancy

Ocular lymphoma is relatively uncommon, accounting for 5–10% of all extranodal lymphomas [288]. However, it is one of the most common orbital malignancies and it is increasing in incidence because of its association with the acquired immunodeficiency syndrome (AIDS) [289]. Ocular lymphoma can be divided into intraocular and adnexal disorders, the former, including malignant lymphoid cells, invade the retina, vitreous body, or optic nerve head; the latter include conditions affecting the eyelid, the conjunctiva, the lacrimal gland, and the orbit [290]. Primary intraocular lymphoma (PIOL) is a subset of primary central nervous system lymphoma. It is usually a large B-cell NHL [291]. PIOL typically presents as a vitritis that is unresponsive to corticosteroid therapy. Diagnosis of PIOL requires pathologic confirmation of malignant cells in specimens of the cerebrospinal fluid, vitreous, or chorioretinal biopsies. The extranodal marginal zone lymphoma (mucosaassociated lymphoid tissue lymphoma) is the dominant lymphoma subtype in the orbit and ocular adnexa. Extranodal marginal zone lymphoma is considered to be the neoplastic counterpart of the marginal zone cells in reactive follicles [292]. Although optimal therapy has yet to be determined [293], it is believed that PIOL should be treated with a combination of chemotherapy and radiation. 

**Figure 5:**
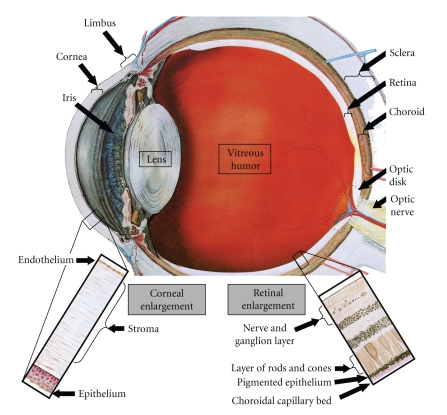
*PPAR*γ* is broadly expressed in the eye providing a pharmacological target for treating ocular angiogenesis.* PPAR*γ* expression is found in the retina including RPE cells, REC, pericytes [287], and ganglion cells. In the cornea, PPAR*γ* is most prominently localized in the epithelial and endothelial layers. Excessive angiogenesis is a pathological hallmark of a number of eye diseases, and anti-VEGF/VEGFR strategies are used therapeutically to treat ocular neovascularization. Manifestations of hematological malignancies in the eye have been documented for leukemia, lymphoma, and multiple myeloma. The potential benefits of PPAR*γ* agonist therapy to inhibit tumor-associated angiogenesis could also be applied to treatment of neovascular eye diseases.

Ocular involvement is common in patients with acute leukemia and has been described in up to half of patients at the time of diagnosis [294]. Eye involvement may be due to leukemic infiltration of various ocular tissues or as a result of one of the secondary complications of the disease [295]. These complications include anemia, thrombocytopenia, and leukostasis, which can lead to retinal hemorrhaging and ischemia [294]. Hemorrhaging in the retina is the most striking feature of ocular leukemia. Furthermore, retinal microaneurysms, capillary closure, and neovascularization have been documented in individuals with chronic leukemia [296, 297]. The treatments include chemotherapy, radiation, or bone marrow transplantation. Ocular findings may be the first manifestation of MM [298]. It may also occur as one of the extramedullary manifestations of the disease or as the first sign of insufficient chemotherapy. MM causes ocular pathology by direct infiltration or as extramedullary plasmacytomas resulting in the displacement or compression of tissues causing hyperviscosity syndrome and immunoglobulin light chain deposition in ocular tissues. Virtually any ocular structure can be affected, including the conjunctiva, cornea, sclera, lens, retina, optic nerve, lacrimal glands, and orbit [298] (Figure 5).

### 3.2. Ocular Neovascularization

Ocular angiogenesis or ocular neovascularization, the abnormal growth of blood vessels in the eye, is the hallmark of the vast majority of eye diseases that cause a catastrophic loss of vision including diabetic retinopathy, AMD, retinopathy of prematurity, and vein occlusion retinopathy [299, 300]. The new vessels may grow into nearly all mature ocular tissue and affect the cornea, iris, retina, and optic disk [301]. They are structurally weak, both leaking fluid and lacking structural integrity. Moreover, the resultant hemorrhage, exudate, and accompanying fibrosis often cause blindness [302].

The cornea is a highly organized transparent tissue located in the anterior part of the eye and it is normally avascular. However, under certain conditions, such as corneal trauma, chemical burns, infection, and inflammation, the development of new blood vessels starts from the vessel of the limbal area (Figure 5). Newly formed blood vessels cover the corneal surface [303], which can lead to severe or permanent visual impairment [302]. The choroid is the layer of blood vessels and connective tissue between the sclera and retina and supplies nutrients to the inner parts of the eye [304]. Choroidal neovascularization (CNV) is associated with many other conditions, such as AMD, inflammatory, infectious, degenerative, hereditary, congenital disorders, tumors, trauma, and a few miscellaneous ocular disorders [302]. In CNV, neovascular channels grow from the choroidal vasculature and extend into the subretinal space leading to local tissue damage. Activation and migration of choroidal ECs (CECs) and retinal pigment epithelial (RPE) cells into the CNV membranes play an important role in the development of the lesion [305]. The mammalian retina is a light sensitive tissue lining the inner surface of the eye, which is composed of multiple cell-types organized within defined layers. It has a dual blood supply from the central retinal artery and the choroidal blood vascular system [304]. Neovascularization of the retina is a critical part of the disease process associated with retinopathy in diabetes, prematurity, and sickle cell disease [302].

### 3.3. Expression of PPAR*γ* in the Eye and Effects on Ocular Neovascularization

PPAR*γ* expression in the mammalian eye has been reported prominently in retina [306, 307] including RPE cells [194, 308, 309], retinal capillary ECs (REC) [310, 311], retinal pericytes [287], and retinal ganglion cells [312]. PPAR*γ* is most prominently localized in the epithelial and endothelial layers of the cornea [198]. PPAR*γ* is also found in CECs [194] and in orbital fibroblasts [313, 314]. The broad expression of PPAR*γ* in the eye provides a pharmacological target for treating ocular angiogenesis.

In vivo alkali-burned mouse cornea experiments showed that neovascularization and scar formation are suppressed by introduction of PPAR*γ* gene expression. PPAR*γ* overexpression suppressed monocyte/macrophage invasion and suppressed the generation of myofibroblasts, as well as upregulation of inflammation/scarring-related growth factors (TGF-*β*, CTGF, and VEGF) and MMPs in a healing cornea. In vitro experiments showed that overexpression of PPAR*γ* suppressed epithelial cell expression of MMP-2/-9 and TGF-*β*1, inhibited cell migration, and suppressed myofibroblast generation upon exposure to TGF-*β*1. Thus, adenoviral-driven expression of the PPAR*γ* gene led to inhibition of the anti-inflammatory and antifibrogenic responses induced in an alkali-burned mouse cornea, and also inhibited activation of ocular fibroblasts and macrophages in vitro [12]. In a VEGF-induced neovascular rat cornea model, intrastromal implantation of the PPAR*γ* ligands pioglitazone [198] or 15d-PGJ_2_ [22] resulted in decreasing MVD, indicating inhibition of ocular angiogenesis. Furthermore, systemic oral administration of rosiglitazone and troglitazone significantly inhibits vessel growth in a dose-dependent fashion in a model of FGF-2-induced mouse corneal neovascularization [195].

PPAR*γ* ligands troglitazone and rosiglitazone inhibit VEGF-induced cell proliferation and migration in bovine CECs and human RPE cells in vitro. Troglitazone also inhibits VEGF-induced tube formation (neovascularization) of CECs [194]. Troglitazone pretreatment can significantly prevent TGF-*β*-induced epithelial-mesenchymal transition of human RPE cells, and retard cell migration [315]. In vivo, laser photocoagulation induced CNV was markedly inhibited by intravitreal injection of troglitazone in rat and monkey eyes. The lesions showed significantly less fluorescein leakage and were histologically thinner in the troglitazone-treated animals without apparent adverse effects in the adjacent retina or in control eyes [194], indicating that the PPAR*γ* ligands are logical for therapy to suppress vascular permeability in the eye. 

PPAR*γ* agonists, troglitazone, rosiglitazone, Pioglitazone, RWJ241947, and 15d-PGJ_2_, inhibit proliferation of human REC and pericytes in vitro through a PPAR*γ*-independent pathway [316]. TZDs downregulate cyclin E (S-phase cyclin) and cyclin A (G2/M-phase cyclin) resulting in cell cycle arrest [316]. Troglitazone and rosiglitazone inhibit VEGF-induced proliferation and tube formation by bovine REC in collagen gels, and inhibit VEGF-induced REC migration in a dose-dependent manner [311]. Retinal angiogenesis is induced in newborn mice by oxygen-induced ischemic injury; however, intravitreal injection of troglitazone or rosiglitazone markedly reduced development of retinal neovascular tissue [311]. In the chick chorioallantoic membrane model of angiogenesis, pioglitazone and rosiglitazone significantly inhibit EC migration as well as the proangiogenic effects of FGF-2 and VEGF [27]. Rosiglitazone may delay the onset of proliferative diabetic retinopathy, possibly because of its antiangiogenic activity [317]. 

Taken together, these studies demonstrate that PPAR*γ* ligands are potent inhibitors of angiogenesis in vivo and in vitro, and suggest that PPAR*γ* may be an important molecular target for inhibiting angiogenesis. The use of PPAR*γ* ligands to prevent pathological angiogenesis holds great potential as a novel therapeutic for neovascularized eye diseases. It may also apply to other neovascularization-related diseases, including hematological malignancies of the eye. However, future clinical investigations should consider analysis of the potential benefits of PPAR*γ* agonist treatment along with ongoing evaluation of potential cardiac risk in studies where the risk-benefit profiles are deemed appropriate [317].

## 4. The Paradox of PPAR*γ* as a Molecular Target in Anticancer Therapy

**Figure 6:**
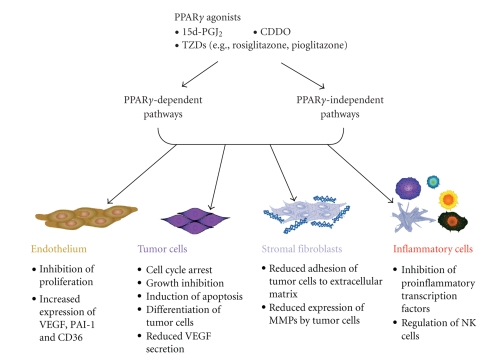
*Direct and indirect effects of PPAR*γ* agonists on tumor and stromal cells.* “Off-target” (PPAR*γ*-independent) effects of PPAR*γ* agonists frequently occur when the agonists are used at high concentrations (much higher than needed to active PPAR*γ* by ligand binding) and in response to electrophilic PPAR*γ* agonists such as 15d-PGJ_2_ and CDDO, which can promote covalent bond formation with cellular proteins in a redox-sensitive manner to modulate signal transduction pathways. PPAR*γ* agonists have been shown to affect almost every stage of tumor progression from inhibition of uncontrolled tumor growth, induction of apoptosis, inhibition of tumor cell adhesion and invasion through stromal compartments into or out of the blood stream, and inhibition of tumor-associated angiogenesis. PPAR*γ* agonists induce expression of tumor-inhibiting molecules such as CD36, the EC receptor for TSP-1, as well as promote the differentiation of tumor cells, which tends to reduce their invasive and metastatic capabilities. The schematic in this figure was adapted from [181].

The aforementioned studies examining the role of PPAR*γ* ligands for treatment of hematological, ocular, and solid malignancies is by no means a complete review of the available literature. The list of off-target effects of PPAR*γ* agonists continues to grow [51]. Furthermore, many of the published studies suggesting that PPAR*γ* ligands exert antitumor properties did not determine whether the effects required ligand activation of the PPAR*γ* transcription factor per se (Table 3). Many human cancer cell lines express high levels of PPAR*γ*, which when treated with high concentrations of TZDs, undergo cell cycle arrest, apoptosis, or differentiation, suggesting a link between PPAR*γ* signaling and their antitumor activities. In contrast, mounting evidence refutes the dependence of the antitumor effects of TZDs on PPAR*γ* activation [25, 51, 318]. Of note, the off-target effects of PPAR*γ* ligands usually occur at much higher concentrations than those required for ligand-dependent PPAR*γ* effects, and there is no correlation between the expression levels of PPAR*γ* in cancer cells and their sensitivity to TZDs [25, 51, 318]. Indeed, PPAR*γ* agonists exert pleiotropic effects on signal transduction pathways involved in cell proliferation, survival and differentiation [25, 51, 71, 188, 318–322] (Table 3 and Figure 6). 

Currently, two PPAR*γ* agonists belonging to the TZDs remain on the market, rosiglitazone (Avandia) and pioglitazone (Actos). In 2000, troglitazone (Resulin) was removed from the market due to severe hepatotoxicity. Moreover, the incidence of delayed drug-induced liver injury that progresses after discontinuation of drug therapy, and whether such injury is specific to just troglitazone or TZDs as a class of drugs, remains unknown [323]. Additional adverse effects associated with TZDs used for insulin sensitizing therapy include edema, weight gain, macular edema, and heart failure [323, 324]. TZDs may cause hypoglycemia when combined with other antidiabetic drugs as well as decrease hematocrit and hemoglobin levels. Furthermore, an increased risk of bone fracture is linked to TZD therapy [324, 325]. When considering the use of PPAR*γ* agonists as adjuvant or combination therapy in hematological malignancies, it will be important to design appropriate preclinical studies that assess the severity of these side effects in the context of each type of cancer. For example, increased edema is associated with increased vascular permeability. The loss of endothelial barrier integrity leads to increased vascular permeability, enhanced transendothelial migration, and metastatic spread of cancer cells [75]. Thus, the potential for TZDs to promote rather than prevent the metastatic spread of cancer should be considered. The malignant proliferation of plasma cells in MM produces skeletal lesions leading to bone pain and pathologic fractures such as vertebral compressions [326]. In that TZDs are associated with increased risk of bone fractures; the use of TZDs for treatment of MM must be evaluated as well. 

Evidence suggesting that the effects of TZDs on improving endothelial-dependent vascular function and decreasing inflammatory biomarkers independently of insulin-sensitizing effects came from studies reporting the effects of TZDs in diabetic and nondiabetic individuals with atherosclerosis [327–329]. In general, PPAR*γ* agonists inhibit tumor-associated angiogenesis by inhibiting FGF-2- and VEGF-induced EC growth, invasion and migration in vitro and in vivo [27, 192], downregulate expression of VEGF by tumor cells [195, 199] and VEGFRs by EC [32], and decrease tumor-associated MVD [24, 32, 198] and EC tube formation [202], measures of angiogenesis in vivo and in vitro, respectively. TZDs inhibit pathological angiogenesis associated with diabetic retinopathy [287, 317], as well as choroidal and retinal neovascularization [194, 198, 311], and suppress primary tumor growth and metastasis by inhibiting angiogenesis [35] (Table 3). Interestingly, in contrast to these reports, TZDs increase VEGF expression in human vascular smooth muscle cells [330] and promote angiogenesis after ischemia [331]. Additional reports suggest that PPAR*γ* ligands are capable of promoting angiogenesis by inducing VEGF expression [28, 30, 203]. 

Huang and colleagues [30] have suggested that pioglitazone has different effects on pathological angiogenesis compared to ischemia-induced collateral vessel growth [332]. TZDs promote differentiation of EPCs/APCs towards the endothelial lineage [197, 200, 201], consistent with the idea that PPAR*γ* ligands have differential effects on angiogenesis needed for restoration of homeostasis in cardiovascular disease or diabetes compared to pathological angiogenesis associated with cancer progression. The role of PPAR*γ* and its ligands in inhibiting or promoting angiogenesis is likely context dependent (Section 2.7 and Table 3) [30, 332]; thus, the use of PPAR*γ* ligands alone or in addition to antiangiogenic agents for treatment of hematological malignancies will require a better understanding of the effects of PPAR*γ* agonists on EC function during pathological angiogenesis.

Many studies have demonstrated beneficial effects of PPAR*γ* agonists on atherosclerosis and ischemia reperfusion injury by reducing inflammation, preventing restenosis after percutaneous coronary intervention, and in some instances, preventing myocardial infarction and cardiovascular death. Recently, however, a number of review articles have discussed the “rosiglitazone debate” about whether taking rosiglitazone puts patients at a higher overall risk of cardiovascular death. The higher risk is based on findings derived from meta-analyses of existing clinical trial data, the release of FDA safety warnings that rosiglitazone increases cardiac ischemic risk, manufacturer updates on TZD labels with a black-box warning for heart failure, as well as warnings and precautions about coadministration of rosiglitazone with nitrate or insulin [333–336]. TZDs are known to induce salt and water retention, which exacerbate the risk of congestive heart failure in patients with type 2 diabetes. Rosiglitazone is a more potent agonist of PPAR*γ* than pioglitazone, thus increased fluid retention and salt imbalance may explain the higher risk of heart failure with this TZD [336]. However, even though treatment with rosiglitazone may, in general, be associated with a higher incidence of cardiovascular events, some studies suggest that there is no increase in all-cause or cardiovascular mortality observed with rosiglitazone treatment [333, 335]. Clearly, prospective randomized trials need to include outcomes measures to determine whether the TZDs and other such compounds under development put patients at a higher overall risk of cardiovascular death. 

As cancer treatments improve, the number of patients who reach the 5-year benchmark of disease-free survival continues to grow. However, adverse effects of anticancer therapy may confound long-term survival. For example, as methods for detecting and treating breast cancer improve, survival of breast cancer patients is increasing but the side effects of adjuvant therapy, including cardiotoxicity, remain clinically important [337]. Agents commonly used for the treatment of breast cancer, including anthracyclines and trastuzumab, have been associated with cardiotoxicity [338], which ranges from subclinical to life-threatening pathology and even fatal results [339]. Imatinib (Gleevec) inhibits the continuously active tyrosine kinase, Bcr-Abl, which results from the translocation of chromosomes 9 and 22 and is effective for the treatment of CML as well as ALL; however, cardiotoxicity is a potentially serious side effect of this drug as well [340]. In that the TZD class of PPAR*γ* agonists is associated with adverse cardiovascular events, additional studies on the efficacy of PPAR*γ* agonists and other lead compounds as adjuvant or combination therapy to treat cancer should be designed to look at the cardiovascular risks and benefits in addition to their efficacy in treating the primary disease.

## 5. Conclusions

The goal to find a cure for all types of cancer is a major initiative of both public and private grant funding institutions and foundations. Thus, forwarding thinking researchers are exploring strategies to identify molecular expression profiles of cancer subtypes and CSCs, to optimize tumor imaging methods to identify cancer micrometastases, as well as to develop more-specific, less toxic drugs through medicinal chemistry to provide tailored therapy to treat and cure cancer in individual patients. However, metastatic disease remains the major cause of morbidity and mortality in both solid tumors and hematological malignancies. Because tumor-associated angiogenesis is critical for cancer progression and metastatic disease, the initiative to identify molecular targets and new or improved chemotherapeutic or biologic agents to inhibit angiogenesis is a high priority area of research in cancer medicine. 

Specific areas of research where PPAR*γ* agonists may be further examined for efficacy in treatment of angiogenesis in hematological malignancies as well as comorbidities that affect quality of life for long-term cancer survivors include signal transduction pathways (e.g., Jak/Stat, PI3K/Akt, PTEN, mTOR) [181, 341, 342], aberrant/oncogenic miRNAs [246, 257, 261, 283, 343–345], targeting CSCs while sparing normal hematopoietic stem cells, and correcting dysregulated metabolic pathways due to drug side effects such as hyperglycemia, hypertension, gastrointestinal toxicity, coagulation disorders, and depression associated with the neurotoxicity of chemotherapeutic drugs [341, 346–348]. Moreover, limitations in the experimental design of published studies should be carefully evaluated. A significant number of studies continue to use troglitazone as a PPAR*γ* agonist despite its having been pulled from the marketplace due to hepatotoxicity. In vitro experiments examining the efficacies of candidate drugs as inhibitors of angiogenesis need to reflect the complexity of the tumor microenvironment in keeping with the in vivo context. For example, large vessel ECs isolated from the veins of human umbilical cords (HUVECs) are frequently used to study angiogenesis by capillary tube formation in 2D-matrix configurations in vitro; however, in vivo tumor-associated angiogenesis occurs in a complex environment composed of multiple cell types including microvessel ECs and matrix constituents in a 3D-configuration. It will also be important to determine whether the therapeutic effects of PPAR*γ* agonists are due to off-target interactions. In conclusion, we hope that this paper has provided a conceptual framework upon which future studies will be designed to unravel the pleiotropic effects of PPAR*γ* in the context of the stromal microenvironment during tumor angiogenesis, growth and metastasis in hematological malignancies.

## Figures and Tables

**Table 1:** PPAR-*γ* ligands.

Natural ligands	Ref.
lysophosphatidic acid	[349]
nitrolinoleic acid	[350]
9-hydroxyoctadecadienoic acid	[351, 352]
13-hydroxyoctadecadienoic acid	[351, 352]
15-hydroxyeicosatetraenoic acid	[353]
prostaglandin D2	[351, 353–357]
15-deoxy-Δ12,14-prostaglandin J_2_ (15d-PGJ_2_)	[351, 353–357]

Synthetic Ligands	Ref

Thiazolidinedione family (TZDs)	[18, 44, 313, 353, 354, 358, 359]
ciglitazone	
pioglitazone	
rosiglitazone	
troglitazone	
TZD 18	
Nonsteroidal anti-inflammatory drugs	[353, 360, 361]
indomethacin	
ibuprofen	
flufenamic acid	
fenoprofen	
L-tyrosine-based	[351, 352]
GW-7845	
GW-1929	
diindolylmethane analogs	[351, 362]
triterpenoid 2-cyano-3,12-dioxooleana-1,9-dien-28-oic acid (CDDO)	[46, 351]
CDDO C-28 methyl ester derivative (CDDO-Me)	[214, 363, 364]
CDCO C-28 imidazole (CDDO-Im)	[50]
1,1-bis[3′-(5-methoxyindolyl)]-1-(*p-t*-butylphenyl) methane (DIM #34),	[365]

**Table 2:** PPAR*γ* and PPAR*γ* ligands as potential therapy for hematological malignancies.

Hematological malignancy/cell line	PPAR*γ* agonist	Comments	Ref
HL-60	troglitazone	Inhibited cell proliferation by G1 arrest; induced differentiation to monocytes	[366]

HL-60	15d-PGJ_2_, troglitazone	Inhibited cell proliferation; induced caspase-dependent apoptosis	[367]

HL-60, K562	15d-PGJ_2_, troglitazone	Induced apoptosis through Bax/Bcl-2 regulation	[368]

Mono Mac 6, U937	15d-PGJ_2_, troglitazone	Induced apoptosis; downregulated cyclooxygenase-2	[369]

HL-60	15d-PGJ_2_	PPAR*γ*-independent TRAIL-induced apoptosis	[370]

Jurkat, PC3	15d-PGJ_2_	PPAR*γ*-independent TRAIL-induced apoptosis	[371]

EoL-1, U937, KPB-M15	troglitazone	Inhibited cell proliferation by G0/G1 arrest	[372]

HL-60, K562	15d-PGJ_2_, troglitazone	Inhibited cell growth, adhesion, and invasion through Matrigel; inhibited MMP-2 and MMP-9 expression	[230]

AML	DIM #34	Inhibited cell growth; induced apoptosis through PPAR*γ*-dependent and independent mechanism	[365]

HL-60, U937, AML, CLL	rosiglitazone, 15d-PGJ_2_, CDDO	Inhibited cell growth, induced differentiation, induced apoptosis when combined with RXR-selective ligands	[373]

HL-60	Thiazolidinedione	Inhibited cell proliferation by G0/G1 arrest; induced apoptosis; induced differentiation	[374]

U937	troglitazone	Inhibited cell proliferation by G1 arrest	[375]

NB4	15d-PGJ_2_, pioglitazone	Inhibited cell proliferation; induced differentiation and lipogenesis when combined with specific RXR ligands	[376]

HL-60, AML	CDDO-Me	Induced cell differentiation; induced apoptosis	[214, 363, 364]

HL-60	CDDO	Induced apoptosis; induced differentiation and increased phagocytosis at sub-apoptotic doses	[377]

APL, NB4, MR2	CDDO	Enhanced all-*trans*-retinoic acid-induced differentiation and apoptosis	[378]

AML	CDDO	Induced apoptosis in a caspase-dependent and independent manner	[379]

U937	CDDO-Im	Inhibited cell proliferation; induced differentiation through PPAR*γ*-independent mechanism	[50]

U937	CDDO, CDDO-Me, CDDO-Im	Induced apoptosis by increasing reactive oxygen species and decreasing intracellular glutathione	[380]

THP-1	rosiglitazone	Inhibited 9-*cis* retinoic acid-induced cell growth	[381]

THP-1	troglitazone, rosiglitazone	Inhibited MCP-1-induced migration	[382]

K562, KU812, KCL22, BV173, SD1, SupB-15	TZD18	Inhibited cell growth through a PPAR*γ*-independent mechanism; inhibited proliferation; induced apoptosis	[359, 383]

K562	troglitazone, pioglitazone	Inhibited cell proliferation and erythroid phenotype; downregulated GATA-1	[384]

B-ALL	15d-PGJ_2_, pioglitazone	Inhibited cell growth by G1 arrest; induced apoptosis partially dependent on caspase signaling	[385]

UTree-O2, Bay91, 380	troglitazone	Inhibited cell growth by G1 arrest; induced apoptosis; downregulates c-myc expression	[386]
U266, RPMI 8226, BL-41, HS-Sultan	15d-PGJ_2_	Induced apoptosis; downregulation of NF-*κ*B-dependent antiapoptotic proteins	[387]

Jurkat, J-Jahn, T-ALL	15d-PGJ_2_, PGD2	Induced apoptosis through PPAR*γ*-dependent mechanism	[388]

Karpas 299	15d-PGJ_2_, GW7845, rosiglitazone	Induced cell death at high ligand concentration but promoted cell survival at low doses	[389]

CTCL and Sezary syndrome cell lines: MJ, Hut78, and HH	CDDO	Induced apoptosis through a PPAR*γ*-independent mechanism by decreasing antiapoptotic protein Bcl-xL and activating caspase 3	[390]

GRANTA-519, Hbl-2, JeKo-1	15d-PGJ_2_, rosi-glitazone, pioglitazone	Induced apoptosis and downregulation of cyclin D1	[391]

CLL B cells	CDDO	Induced apoptosis in part by activation of caspase-8	[392]

CLL B cells, Jurkat	CDDO	Induced apoptosis through the intrinsic pathway	[393]

DLBCL	CDDO	Inhibited proliferation; induced apoptosis through a PPAR*γ*-independent mechanism	[47]

Primary B lymphocytes, Ramos, OCI-Ly19 DLBCL	CDDO, CDDO-Im, Di-CDDO	Induced apoptosis through a mitochondrial dependent pathway	[394]

ANBL6, RPMI 8226	15d-PGJ_2_, ciglitazone	Induced apoptosis via caspase activation and mitochondrial depolarization	[208]

LP-1, U-266, RPMI 8226-S, OPM-2, IM-9	rosiglitazone, pioglitazone, 15d-PGJ_2_	Inhibited tumor cell growth	[395]

Waldenstrom's macroglobulinemia	rosiglitazone, ciglitazone	Inhibited cell growth; induced apoptosis	[396]

multiple myeloma (MM) drug sensitive MM.1S or drug resistant MM.1R cells, KAS6/1, ANBL-6	15d-PGJ_2_, troglitazone	Inhibited cell adhesion to BMSCs and adhesion-triggered IL-6 production; overcame resistance to dexamethasone (MM.1R cells)	[212]

MM cells, U266, RPMI 8226, bone marrow mononuclear cells	CDDO, CDDO-Im	Induced apoptosis by disruption of mitochondrial membrane potential	[397]

Dexamethasone-resistant MM.R1, RPMI 8226/LR-5, RMPI 8226/Dox-40, U266	CDDO-Im	Induced apoptosis; decreased MM adhesion-triggered IL-6 production	[398]

RPMI 8226, JJN3	CDDO-Im	Inhibited Stat3 and Stat5 phosphorylation; induced Stat inhibitors SOCS-1 and SHP-1	[399]

Normal human B cells and B lymphoma cells (Daudi, Ramos, Raji)	rosiglitazone, pioglitazone, 15d-PGJ_2_	Inhibited cell proliferation; induced apoptosis	[209]

MM cell lines (RPMI 8226 and U266); BMSCs, HS-5	PPAR*γ* over-expression; ciglitazone	PPAR*γ* overexpression inhibited proliferation and induced apoptosis in MM cells; inhibited IL-6 production in BMSCs	[207]

B cell lymphoma (Raji, Ramos cell lines)	PPAR*γ* siRNA	Silencing of PPAR*γ* induced cell proliferation and cell differentiation; PPAR*γ* knockdown enhanced NF-*κ*B activity in Ramos cells	[206]

**Table 3:** Effects of PPAR*γ* agonists on endothelial cell function and angiogenesis.

Ref.	Goal of Study	Results	Pro- or antiangiogenic	Direct or Indirect Effects
[192]	To determine whether PPAR*γ* ligands induce EC proliferation or influence cytokine-induced proliferation in vitro.	PPAR*γ* ligands troglitazone and pioglitazone negligibly affected basal EC proliferation in vitro; troglitazone and pioglitazone significantly inhibited FGF-2-induced EC growth.	• Antiangiogenic activity as shown by inhibiting FGF-2-induced EC proliferation	Not reported

[22]	To determine effects of PPAR*γ* ligands on in vitro and in vivo angiogenesis and EC proliferation.	15d-PGJ_2_, BRL49653, or ciglitazone, dose-dependently suppresses HUVEC differentiation into tube-like structures and cell proliferation; 15d-PGJ_2_ downregulated VEGFR1, VEGFR2 and uPA and increased PAI-1 mRNA expression in vitro; 15d-PGJ_2_ inhibited angiogenesis in vivo.	• Antiangiogenic activity	Not reported
			• Anti-cell proliferation and anti-cell differentiation activity	

[193]	To determine whether human ECs express PPAR*γ* and if PPAR*γ* regulates PAI-1 expression in EC.	ECs expressed functionally active PPAR*γ*; PPAR*γ* ligands (15d-PGJ_2_) and oxidized linoleic acid regulated PAI-1 expression in ECs.	• Antiangiogenic activity by inhibiting fibrinolysis (fibrin induces angiogenesis)	Not reported

[194]	To determine the antiangiogenic effects of PPAR*γ* agonists on CNV in vitro and on experimental laser photocoagulation-induced CNV in vivo.	PPAR*γ* ligands troglitazone and rosiglitazone inhibited VEGF-induced migration and proliferation of human RPE cells and bovine CECs and tube formation of CEC in a dose-response manner; troglitazone inhibited CNV in rat and monkey eyes.	• Antiangiogenic activity in the eye	Not reported
			• Anti-cell proliferation activity	

[195]	To determine whether PPAR*γ* ligands inhibit cancer cell growth and cancer-associated angiogenesis.	PPAR*γ* expressed in tumor EC; rosiglitazone suppressed primary tumor growth and metastasis; rosiglitazone inhibited bovine capillary EC but not tumor cell proliferation; rosiglitazone decreased VEGF production by tumor cells in vitro; rosiglitazone suppressed angiogenesis in vivo and in a variety of primary tumors.	• Antiangiogenic activity	Direct and indirect
			• Anti-EC but not tumor cell proliferation activity	

[196]	To determine whether PPAR*γ* ligands regulate PPAR*γ* and CD36 gene expression in microvascular and large vessel EC in vitro and modulate TSP-1 peptide ABT510 antiangiogenic activity in tumor-associated endothelium in vivo (mouse tumor models).	15d-PGJ_2_, troglitazone, and rosiglitazone induced PPAR*γ* and CD36 gene expression in EC in vitro and inhibited angiogenic endothelial functions in vitro and neovascularization in vivo in an additive manner; ABT510 and PPAR*γ* ligands enhancedsynergistically the antiangiogenic and antitumor effects of TSP-1 peptide ABT510.	• Antiangiogenic activity	Direct for in vitro activities
			• Anti-proliferation activity in EC	
			• Anti-invasion activity of EC	
			• Cooperative inhibition of EC angiogenic functions	
			• Synergistic inhibition of tumor angiogenesis	

[197]	To determine whether PPAR*γ* agonists modulate bone marrow-derived bipotential APCs to promote endothelial lineage differentiation and re-endothelialization after vascular intervention.	Rosiglitazone promoted differentiation of bone marrow-derived APCs toward the endothelial lineage and attenuated restenosis after angioplasty in C57/BL6 mice; rosiglitazone inhibited APC differentiation toward smooth muscle cell lineage.	• Proangiogenic activity	Not reported
			• Anti-inflammatory	
			• Promoted lineage-specific differentiation	

[198]	To determine the efficacy of pioglitazone to inhibit corneal neovascularization.	PPAR*γ* ligand pioglitazone decreased MVD in a VEGF-induced neovascularization in a rat cornea model.	• Antiangiogenic activity in the eye	Not reported

[24]	To determine whether PPAR*γ* ligands can inhibit angiogenesis in A549 lung cancer cell xenograft in vivo and which signaling pathway is involved in vitro.	PPAR*γ* ligands troglitazone and pioglitazone significantly inhibited A549 primary tumor growth in SCID mice, likely due to inhibition of cancer-associated angiogenesis; in vitro studies on A549 cells suggested PPAR*γ* ligands inhibit chemokine expression and inhibit NF-*κ*B activity, the transcription factor necessary for chemokine expression.	• Antiangiogenic activity	Direct and indirect
			• Inhibited NF-*κ*B transcription factor activity	

[199]	To determine effects of PPAR*γ* ligands on VEGF expression by human endometrial cells.	PPAR*γ* ligands rosiglitazone and 15d-PGJ_2_ repressed VEGF gene expression through a PPRE in the VEGF promoter.	• Antiangiogenic activity	Not reported
			• Identified PPRE in VEGF promoter	

[200]	Because endothelial precursor cell (EPC) function is impaired in type 2 diabetic patients and EC dysfunction can be ameliorated by treatment with TZDs, this study asked whether TZDs affect the number and function of EPCs.	Rosiglitazone improved number and migratory activity of EPCs from type 2 diabetic patients; rosiglitazone increased the CD133+ subpopulation of CD34+ cells (stem cells); rosiglitazone increased circulating levels of VEGF; effects may be due to increased bioavailability of NO by Akt-dependent phosphorylation of eNOS—a pathway that is activated by VEGF or the insulin signaling cascade.	• Proangiogenic activity	Not reported
			• Akt survival pathway activated	
			• Elevated CD133+/CD34+ stem cells towards EC lineage (VE-cadherin+ and CD31+)	

[201]	To determine whether TZDs increase the number of bone marrow-derived EPCs in mice and the signaling pathways activated.	Treatment of mice with pioglitazone upregulated bone marrow and circulating EPCs; pioglitazone prevented apoptosis of human and mouse EPCs in a PI3K-dependent manner in vitro.	• Proangiogenic activity	Not reported; indirect activation of PI3K-Akt not activated by pioglitazone
			• PI3K activated	
			• Anti-apoptotic	

[27]	To study the effect of PPAR*γ* agonists on VEGF- and FGF-2-induced angiogenesis and EC migration.	Pioglitazone and rosiglitazone inhibited the proangiogenic effects of FGF-2 and VEGF in the chick chorioallantoic membrane model angiogenesis; pioglitazone and rosiglitazone inhibited VEGF- and FGF-2-induced EC migration.	• Antiangiogenic in vivo	not reported
			• Inhibited EC migration	

[28]	To determine whether activation of PPAR*α* and PPAR*γ* stimulates angiogenesis.	PPAR*α* agonist WY14643 and PPAR*γ* agonist GW1929 induced EC tube formation in EC/interstitial cell cocultures by increasing VEGF production; WY14643 and GW1929 induced angiogenesis in murine corneal angiogenesis model and Akt activated in vitro.	• Proangiogenic activity	Direct for both PPAR*α* and PPAR*γ*
			• Induced VEGF production	
			• Prosurvival	

[30]	To investigate the impact of diabetes on ischemia-induced collateral vessel growth, and tested the hypothesis that PPAR*γ* agonists augment collateral flow to ischemic tissue.	Pioglitazone ameliorated endothelial dysfunction and enhanced blood flow recovery after tissue ischemia in diabetic mice; pioglitazone restored VEGF levels that were reduced by ischemic injury; Activation of eNOS essential for pioglitazone to promote angiogenesis in ischemic tissue.	• Proangiogenic activity	Not reported
			• Induced VEGF production	

[202]	To determine effects of rosiglitazone on gastric cancer cell cycle, proliferation, migration, and invasion; endothelial capillary tube formation (an in vitro measure of angiogenesis).	Rosiglitazone inhibited gastric cancer cell growth, caused G1 cell cycle arrest and induced apoptosis in a dose-dependent and PPAR*γ*-dependent manner; rosiglitazone inhibited gastric cancer cell migration, invasion, and expression of MMP-2 in a dose-dependent manner in a PPAR*γ*-independent manner; rosiglitazone reduced VEGF-induced “angiogenesis” of HUVEC in a dose- and PPAR*γ*-dependent manner.	• Antiangiogenic activity	Not reported
			• Antitumor cell proliferation activity	
			• Anti-invasion	
			• Proapoptotic	

[32]	To determine the effects of PPAR*γ* ligands on pancreatic cancer-associated angiogenesis, VEGF expression, and tumor growth in vitro and in vivo.	Rosiglitazone inhibited pancreatic carcinoma growth both in vitro and in vivo; rosiglitazone suppressed xenograft tumor angiogenesis by downregulating VEGF expression; 15d-PGJ_2_, 9-cis-RA, and their combination inhibited VEGF mRNA expression in PANC-1 cells in a dose- and time-dependent manner; MVD was decreased in rosiglitazone-treated mice.	• Antiangiogenic activity	Not reported
			• Antitumor cell proliferation activity	

[203]	To determine whether adipose tissue angiogenesis was stimulated by rosiglitazone using an assay to study angiogenic sprout formation ex vivo.	Obesity and TZD treatment in vivo induced angiogenic sprout formation from adipose tissue fragments, but not from aorta rings; rosiglitazone induced expression of VEGF-A, VEGF-B, and ANGPTL4; ANGPTL4 stimulated EC growth and capillary tube formation; ANGPTL4 alleviated the growth inhibitory actions of rosiglitazone on ECs in the presence or absence of VEGF likely causing a net expansion of the capillary network in adipose tissue in response to PPAR*γ* activators.	• Proangiogenic activity in adipose tissue	Indirect likely via a PPAR*γ*-stimulated adipocyte-specific factor ANGPLT4 capable of overcoming direct antiangiogenic effect of rosiglitazone on ECs
			• Induced VEGF production	
			• Induced ANGPLT4 expression	

## Abbreviations

15d- PGJ_2_: 15-deoxy-Δ-12-14-prostaglandin J_2_
Ago: Argonaute
AIDS: Acquired immunodeficiency syndrome
Akt/PKB: v-akt murine thymoma viral oncogene homolog/protein kinase B
AMD: Age-related macular degeneration
AML: Acute myeloid leukemia
ANGPTL4: Angiopoietin-like factor-4
AP-1: Activator protein 1
APC: Angiogenic precursor cell
APL: Acute promyelocytic leukemia
ARNT: Aryl hydrocarbon nuclear translocator
B-ALL: B type acute lymphoblastic leukemia
Bcr-Abl: Breakpoint cluster region-Abelson murine leukemia viral oncogene homolog 1/Philadelphia chromosome
BMSC: Bone marrow stromal cell
C/EBP: CAAT enhancer binding protein
CAM: Chorioallantoic membrane
CDDO-Im: CDDO C-28 imidazole
CDDO-Me: CDDO C-28 methyl ester derivative
CDDO: 2-cyano-3,12-dioxooleana-1,9-dien-28-oic acid
CEC: Choroidal endothelial cell
CLL: Chronic lymphocytic leukemia
CML: Chronic myeloid leukemia
CNV: Choroidal neovascularization
CSC: Cancer stem cell
CTCL: Cutaneous T cell lymphoma
CTGF: Connective tissue growth factor
DIM #34: 1,1-bis[3′-(5-methoxyindolyl)]-1-(p-t-butylphenyl) methane
DLBCL: Diffuse large B cell lymphoma
EC: Endothelial cell
ECM: Extracellular matrix
EPC: Endothelial precursor cell
FAT: Fatty acid translocase
FGF-2: Fibroblast growth factor-2
gp130: Glycoprotein 130
HIF: Hypoxia inducible factor
HSF: Hepatocyte stimulatory factor
HSPG: Heparan sulfate proteoglycan
HUVEC: Human umbilical vein endothelial cell
IGF-1: Insulin-like growth factor
I*κ*B: Inhibitor of *κ*B
LDL: Low density lipoprotein
MAPK: Mitogen-activated protein kinase
MASPIN: Mammary serine protease inhibitor (tumor suppressor gene)
MCP-1/CCL2: Macrophage chemotactic protein
MIF: Macrophage inhibitory factor
miRNA: MicroRNA
MM: Multiple myeloma
MMEC: Multiple myeloma derived endothelial cell
MMP: Matrix metalloproteinase
mTOR: Mammalian target of the rapamycin
MVD: Microvessel density
NF-*κ*B: Nuclear factor *κ*B
NHL: Non-Hodgkin lymphoma
NOD/SCID: Nonobese diabetic/severe combined immune deficiency
NRP: Neuropilin
p300/CBP: Transcriptional coactivator protein/cAMP-response element-binding protein (CREB) binding protein
PAI: Plasminogen activator inhibitor
PDGF: Platelet derived growth factor
PGC-1: PPAR*γ* coactivator-1
PI3K: Phosphatidylinositol 3-kinase
PIOL: Primary intraocular lymphoma
PLGF: Placenta growth factor
PPAR: Peroxisome proliferator-activated receptor
PPRE: PPAR*γ* response element
PTEN: Phosphatase and tensin homolog (tumor suppressor gene)
pVHL: Protein von Hippel-Lindau
REC: Retinal capillary endothelial cell
RISC: RNA induced silencing complex
RPE: Retinal pigmented epithelial
SBE: Stat3 Binding Element
SMRT/NCoR: Silencing mediator for retinoid and thyroid hormone receptors/nuclear receptor corepressor
SOCS: Suppressor of cytokine signaling
Src: v-src sarcoma (Schmidt-Ruppin A-2) viral oncogene homolog (avian); aka, p60-Src
STAT: Signal transducer and activator of transcription
TGF-*α**/**β**:*: Transforming growth factor
TIMP: Tissue inhibitor of metalloproteases
TNF-*α**:*: Tumor necrosis factor
TPM1: Tropomysin 1 (tumor suppressor gene)
Tro: Troglitazone
TSP: Thrombospondin
TZDs: Thiazolidinediones
UTR: Untranslated region
VEGF: Vascular endothelial growth factor
VEGFR: VEGF receptor
VPF: Vascular permeability factor.
